# Discovery of Indoleamine 2,3-Dioxygenase 1 (IDO-1) Inhibitors Based on *Ortho*-Naphthaquinone-Containing Natural Product

**DOI:** 10.3390/molecules24061059

**Published:** 2019-03-18

**Authors:** Hongchuan Zhao, Pu Sun, Wei Guo, Yi Wang, Ao Zhang, Linghua Meng, Chunyong Ding

**Affiliations:** 1 CAS Key Laboratory of Receptor Research, State Key Laboratory of Drug Research, Shanghai Institute of Materia Medica, Chinese Academy of Sciences, Shanghai 201203, China; 201628012342026@simm.ac.cn (H.Z.); aozhang@simm.ac.cn (A.Z.); 2 Division of Anti-Tumor Pharmacology, Shanghai Institute of Materia Medica, Chinese Academy of Sciences, Shanghai 201203, China; jnsunpu@126.com (P.S.); wguo@jding.dhs.org (W.G.); yiwang@simm.ac.cn (Y.W.); 3 School of Pharmacy, University of Chinese Academy of Sciences, Beijing 100049, China; 4 School of Life Scienece and Technology, ShanghaiTech University, Shanghai 20120, China

**Keywords:** indoleamine 2,3-dioxygenase 1, immunosuppressive, natural product, *ortho*-naphthaquinone, molecular docking

## Abstract

There is great interest in developing small molecules agents capable of reversing tumor immune escape to restore the body’s immune system. As an immunosuppressive enzyme, indoleamine 2,3-dioxygenase 1 (IDO-1) is considered a promising target for oncology immunotherapy. Currently, none of IDO-1 inhibitors have been launched for clinical practice yet. Thus, the discovery of new IDO-1 inhibitors is still in great demand. Herein, a series of diverse *ortho*-naphthaquinone containing natural product derivatives were synthesized as novel IDO-1 inhibitors. Among them, 1-ene-3-ketone-17-hydroxyl derivative **12** exhibited significantly improved enzymatic and cellular inhibitory activity against IDO-1 when compared to initial lead compounds. Besides, the molecular docking study disclosed that the two most potent compounds **11** and **12** have more interactions within the binding pocket of IDO-1 via hydrogen-bonding, which may account for their higher IDO-1 inhibitory activity.

## 1. Introduction

Although immune checkpoint blockades such as anti-PD-L1 (programmed cell death-ligand 1), anti-PD-1 (programmed cell death protein 1) [1], and anti-CTLA4 (cytotoxic T-lymphocyte-associated protein 4) [2] have demonstrated attractive therapeutic effects in multiple clinical trials, this new modality often suffers from a low response rate at least due to the immune escape developed by tumors [3]. Therefore, there is great interest in developing small molecule agents capable of reversing tumor immune escape to restore the body’s immune system.

**Figure 1:**
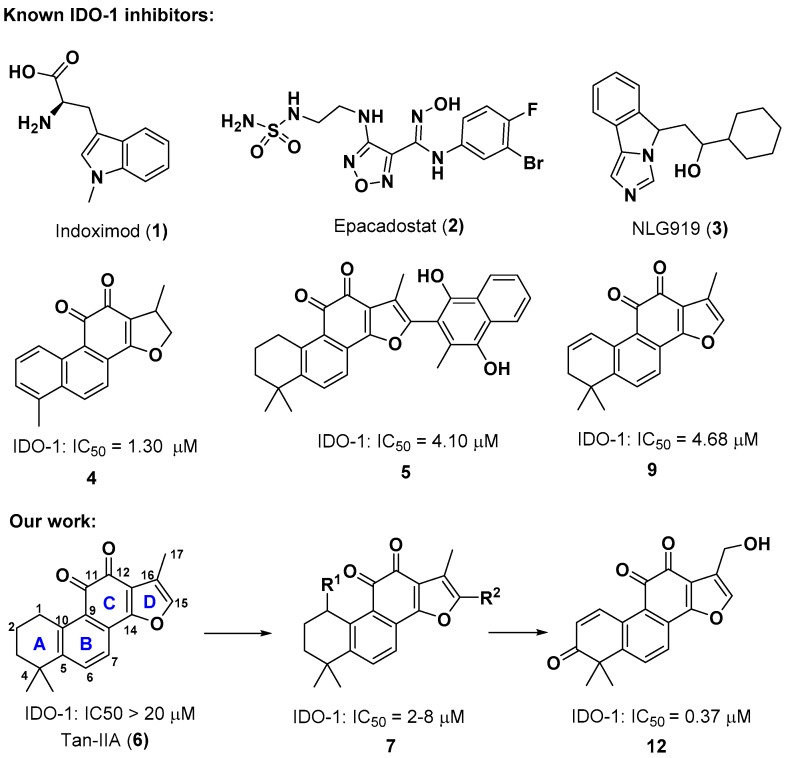
Structures of known and our reported indoleamine 2,3-dioxygenase 1 (IDO-1) Inhibitors.

Indoleamine 2,3-dioxygenase 1 (IDO-1) is a monomeric heme-containing enzyme found in nonhepatic human tissues [4]. It catalyzes the oxidative cleavage of the pyrrole ring of l-tryptophan (l-Trp) in the first and rate-limiting step of the kynurenine pathway to produce *N*-formylkynurenine [5]. This reaction not only leads to a local decrease of l-Trp concentration, but also generates a variety of catabolic products, which both account for the immunosuppressive effects of IDO-1 [6,7,8,9]. Thus, IDO-1 is considered as one of the key factors in the process of immune evasion in tumor microenvironment [10]. Overexpression of IDO-1 has been observed in a number of human malignancies, including ovarian, colorectal, and pancreatic cancers [11]. A growing body of clinical evidence indicated that the high expression of IDO-1 in tumors is directly correlated with a low survival rate [12]. In addition, IDO-1 inhibitors have demonstrated a significant cooperative effect with chemotherapy, radiotherapy, or cancer vaccines in preclinical models of cancer [13,14,15,16]. Accordingly, inhibition of IDO-1 would be a very useful strategy for the treatment of cancer. In the past few years, intense efforts have been devoted to developing IDO-1 inhibitors for the immuno-oncology therapy [17]. A number of structurally diverse natural products and synthetic IDO-1 inhibitors have been reported. Several IDO-1 inhibitors entered clinical trials such as indoximod (**1**) [13,18], epacadostat (**2**) [3], and NLG919 (**3**) (Figure 1) [19]. However, none of them have been launched for clinical practice yet. Thus, the discovery of new IDO-1 inhibitors is still in great demanded.

Natural products have long served as valuable starting points for drug discovery due to their unique molecular frameworks and novel mechanisms of actions. Danshen, a well-known traditional Chinese medicine (TCM) herb derived from the dried root or rhizome of *Salvia miltiorrhiza Bunge*, has long been used in Asian countries for multiple therapeutic remedies including cardiovascular and cerebrovascular disorders as well as inflammatory diseases [20,21,22]. Tanshinones such as Tan-IIA (**6**) (Figure 1), a group of lipophilic furano-*o*-naphthaquinone diterpenes isolated exclusively from this TCM herb, have demonstrated various pharmacological activities, such as cardio-protection, antibacterial, anti-inflammatory, antioxidant, anti-platelet aggregation, and anticancer properties [23,24]. 

As part of our drug discovery program towards identification of lead compounds from natural products bearing *o*-naphthaquinone scaffold [25,26,27,28,29,30,31], we screened our in-house compound library derived from *o*-naphthaquinones to pursue novel IDO-1 inhibitors, and found acyloxy derivatives of **6** possessing moderate IDO-1 inhibitory activity. When our project was ongoing, three *o*-naphthaquinone derivatives **4**, **5**, **9** were also identified as IDO-1 inhibitors by Wang and Xu et al. using machine-learning-based virtual screening (Figure 1) [32]. Inspired by these results, we further performed structural optimization based on the acyloxy derivatives to explore the chemical space of *o*-naphthaquinone scaffold for IDO-1 inhibition. Herein, we disclosed our effort on the chemical synthesis, biological evaluation, and molecular docking of *o*-naphthaquinone derivatives as novel IDO-1 inhibitors.

## 2. Results and Discussion

### 2.1. Chemistry

Following our previously established acyloxylation procedure [26,27,28], a series of diverse acyloxy derivatives **7a**–**m** [27] were readily accessed by action of different carboxylic acids with **6** in 45%–95% yields (Table 1).

**Scheme 1:**
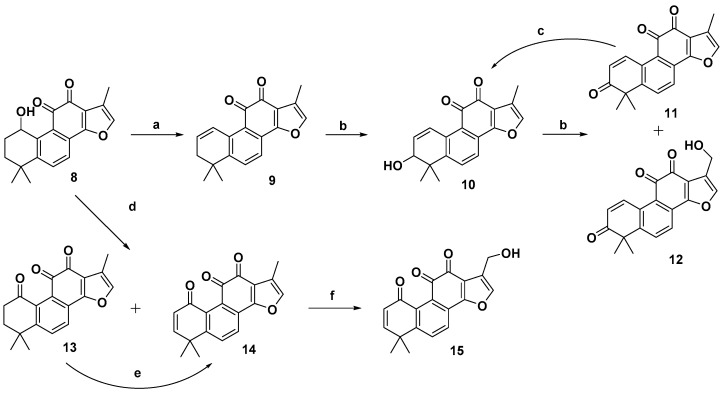
Synthesis of *o*-naphthaquinone derivatives **11**–**15**. Reagents and conditions: (**a**) PPTS, toluene, 110 °C, 12 h; (**b**) SeO_2_, 1,4-dioxane, 100 °C; (**c**) NaBH_4_, MeOH, rt; (**d**) IBX, toluene/DMSO = 4:2, 60 °C, microwave, 3 h; (**e**) DDQ, toluene, 80 °C, 72 h; (**f**) SeO_2_, 1,4-dioxane, 100 °C, 36 h.

As outlined in Scheme 1, further hydrolysis of compound **7a** with K_2_CO_3_ in methanol afforded 1-hydroxyl product **8** [33] in 99% yield, which further underwent an elimination reaction in the presence of pyridinium *p*–toluenesulfonate (PPTS) at 110 °C to give the 1-ene analogue **9** [33] in 85% yield. To introduce a hydroxyl group to the 3-position of the A-ring, an allylic oxidation was performed by treatment of **9** with selenium dioxide in refluxing 1,4-dioxane/H_2_O for 1.5 h to directly produce the Δ^1^-3-ketone (1-ene-3-ketone) derivative **11** [34,35] in 81% yield as sole product, instead of the 1-ene-3-hydroxyl derivative **10**. Interestingly, prolonged reaction time not only provided **11**, but also gave rise to 17-hydroxyl enone derivative **12** in 28% yield. It was believed that compounds **11** and **12** were produced by further oxidation of **10** although it was not observed in the reaction. Alternatively, reduction of **11** with NaBH_4_ provided allylic alcohol **10**. In addition, treatment of compound **8** with 2-iodoxybenzoic acid in a mixture solvent of toluene and DMSO produced 1-ketone **13** [33] in 27% yield together with some minor enone derivative **14**. Further oxidation of **13** with 2,3-dichloro-5,6-dicyano-1,4-benzoquinone (DDQ) could readily produce 1-ketone-Δ^2^ (1-ketone-2-ene) compound **14** in 70% yield. Subsequently, refluxing of **14** in dioxane/H_2_O using SeO_2_ as the oxidant afforded 17-hydroxy enone derivative **15** as the single product.

### 2.2. Biological Evaluation

All the synthesized derivatives were evaluated for their IDO-1 inhibitory activities using epacadostat as the positive control agent. The potent compounds were further assayed for their cellular activity in HEK 293 cells over-expressing human IDO-1. As shown in Table 2, a series of acyloxylated derivatives **7a**–**m** was first evaluated. Compared to **6**, acyclic acyloxylated derivatives **7a**–**b** and **7e**–**f** displayed significantly improved IDO-1 inhibitory activity with IC_50_ values ranging from 2 to 8 µM. However, installation of aryl or heteroaryl group at C-15 of the furan ring (**7c**–**d**) resulted in loss of the activity with IC_50_ value greater than 20 µM. Among cyclic acyloxylated derivatives **7g**–**m**, furan compound **7k** possessed the most potent activity against IDO-1 with an IC_50_ value of 4.60 µM. Most of the six-membered cyclic acyloxylated derivatives **7h**–**i** exhibited relatively weak activity with IC_50_ values around 20 µM except pyrazine compound **7m**. Hydrolysis of the acyloxyl into the hydroxyl (compound **8**) displayed comparable inhibitory activity to that of precursor **7a**. Compound **9** with 1-ene possessed 61.2% inhibitory rate at 20 µM, which is less potent than that reported by literature [32]. Compound **10** with hydroxyl group at the allylic position recovered the activity with an IC_50_ value of 3.10 µM. Compound **11** with 1-ene-3-ketone moiety exhibited significantly improved IDO-1 inhibitory activity with an IC_50_ value of 0.84 µM, and 17-hydroxyl derivative **12** also possessed more potent activity with 0.37 µM IC_50_ value. By contrast, both 1-ketone-2-ene compound **14** and its 17-hydroxyl analogue **15** demonstrated weak activities with IC_50_ values around 20 µM.

Compounds **9**–**14** were further assayed for their activity to inhibit IDO-1 in HEK 293-hIDO-1 cells. Compared to their enzymatic activity, most of them displayed less potent inhibitory activity against IDO-1 probably due to their poor permeability. Among them, compound **12** exhibited the most potent cellular inhibitory activity with an IC_50_ value of 3.85 µM. The calculated cLogP values of **11** and **12** with Chemdraw were 3.90 and 2.36, respectively, which may account for their difference on the cellular potency.

### 2.3. Molecular Docking Study

**Figure 2:**
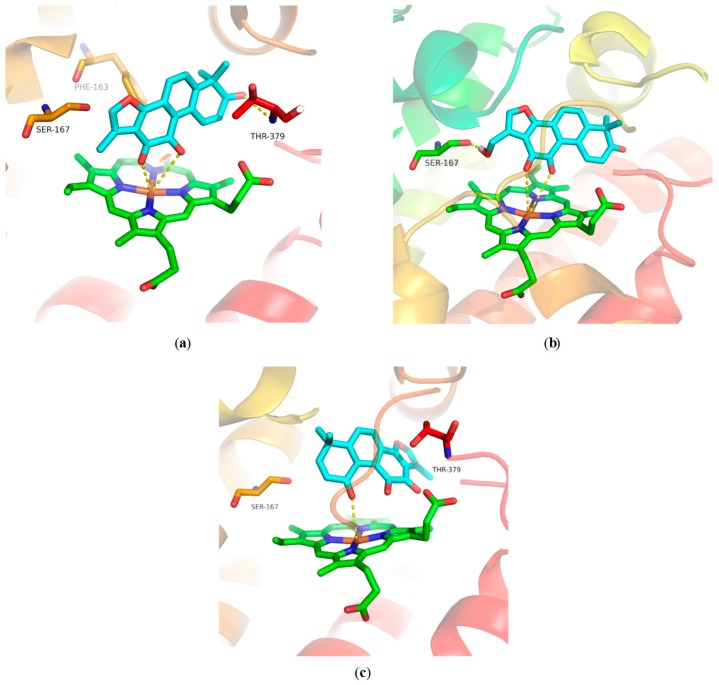
Docking models of compounds **11**, **12,** and **14** bound to IDO-1. (**a**) Computational modeling at the active site of IDO-1 for **11**; (**b**) computational modeling at the active site of IDO-1 for **12**; (**c**) computational modeling at the active site of IDO-1 for **14**.

To understand the binding modes of our compounds for IDO-1, compounds **11**, **12**, and **14** were docked into the binding pocket of an IDO-1 X-ray crystal structure (PDB code 5WHR). All of them could interact with residues Phe226, Thr379, Phe163, Tyr126, and Gly378 within hydrophobic pockets (Figure S17). The *ortho*-diketone moiety of **11** interacted with heme Fe^2+^ through two coordination bonds, and its 3-ketone had an additional hydrogen-bond interaction with Thr379 (Figure 2a). For compound **12**, the *ortho*-diketone moiety not only formed two coordination bonds with heme Fe^2+^ (Figure 2b), but also had a hydrogen-bond interaction with the NH of Ala264 (Figure S17b). Besides, the 17-hydroxyl group of **12** also interacted with residue Ser167 via a hydrogen-bond (Figure S17b). The 1-ketone group of **14** formed only one coordination bond with Fe^2+^ of heme (Figure 2c). These docking results suggested that compounds **12** and **11** have more interactions with IDO-1 than **14**, which may account for their more potent IDO-1 inhibitory activity.

## 3. Materials and Methods 

### 3.1. Chemistry

#### 3.1.1. General Chemistry

All solvents and chemical reagents were obtained from commercial sources and used without further purifications. ^1^H- and ^13^C-NMR spectra were recorded on a Varian Mercury 400 NMR spectrometer (Varian, San Francisco, CA, USA) and Bruker avance III (Bruker, Karlsruhe, Germany). High-resolution mass spectrometry (HRMS) analysis was recorded at an ionizing voltage of 70 eV on a Agilent G6520 Q-TOF spectrometer (Agilent, San Francisco, CA, USA). Flash column chromatography on silica gel (200–300 mesh) was used for the routine purification of reaction products. All reactions were monitored by TLC on silica gel plates.

#### 3.1.2. Procedure for the Preparation of Compounds

##### Synthesis of 1,6,6-trimethyl-6,7-dihydrophenanthro[1,2-b]furan-10,11-dione (**9**) [33]

To a solution of **8** (100 mg, 0.322 mmol) in toluene (3 mL) was added pyridinium *p*–toluenesulfonate (PPTS, 248 mg, 0.483 mmol). The reaction mixture was stirred at 110 °C for 12 h and then diluted with H_2_O and extracted with EtOAc (100 mL × 3). The combined organic layer was washed with brine, dried over anhydrous Na_2_SO_4_, and concentrated in vacuo. The residue was further purified by silica gel column, and elution with 15%–10% EtOAc in hexane afforded the desired product **9** (80 mg, 85%) as a red solid. ^1^H-NMR (400 MHz, CDCl_3_) δ 7.85 (dt, *J* = 10.2, 1.8 Hz, 1H), 7.55 (s, 2H), 7.23 (d, *J* = 1.3 Hz, 1H), 6.33 (dt, *J* = 10.1, 4.6 Hz, 1H), 2.29 (d, *J* = 1.8 Hz, 1H), 2.28 (d, *J* = 1.9 Hz, 1H), 2.27 (d, *J* = 1.3 Hz, 3H), 1.30 (s, 6H). ^13^C-NMR (126 MHz, CDCl_3_) δ 184.4, 175.9, 161.5, 148.5, 141.3, 137.4, 134.0, 130.3, 127.3, 124.5, 123.1, 121.5, 121.1, 120.0, 37.9, 34.2, 28.4, 8.8.

##### Synthesis of 1,6,6-trimethylphenanthro[1,2-b]furan-7,10,11(6*H*)-trione (**11**)

To a solution of **9** (35 mg, 0.120 mmol) in 1,4-dioxane (2 mL) was added selenium dioxide (13 mg, 0.120 mmol). The reaction mixture was stirred at 100 °C for 1.5 h and then diluted with H_2_O and extracted with EtOAc (50 mL × 3). The combined organic layer was washed with brine, dried over anhydrous Na_2_SO_4_, and concentrated in vacuo. The residue was further purified by silica gel column, and elution with 1% MeOH in CH_2_Cl_2_ afforded the desired product **11** (30 mg, 82%) as a red solid. m.p. 261.2–262.8 °C. ^1^H-NMR (400 MHz, CDCl_3_): δ 8.99 (d, *J* = 10.5 Hz, 1H), 7.79 (d, *J* = 8.2 Hz, 1H), 7.72 (d, *J* = 8.1 Hz, 1H), 7.30 (s, 1H), 6.41 (d, *J* = 10.5 Hz, 1H), 2.29 (s, 3H), 1.51 (s, 6H). ^13^C-NMR (126 MHz, CDCl_3_) δ 201.9, 184.2, 175.3, 160.5, 150.7, 142.0, 139.2, 132.3, 132.3, 128.6, 128.5, 125.3, 123.5, 121.5, 120.6, 47.9, 27.6, 8.8. MS (ESI, [M + Na]^+^) *m*/*z* 329.3. HRMS (ESI, [M + H]^+^) calcd for C_19_H_15_O_4_, 307.0965; found, 307.0973.

##### Synthesis of 1-(hydroxymethyl)-6,6-dimethylphenanthro[1,2-b]furan-7,10,11(6*H*)-trione (**12**)

To a solution of **9** (80 mg, 0.273 mmol) in 1,4-dioxane (2 mL) was added selenium dioxide (30 mg, 0.273 mmol). The reaction mixture was stirred at 100 °C for 24 h and then diluted with H_2_O and extracted with EtOAc (100 mL × 3). The combined organic layer was washed with brine, dried over anhydrous Na_2_SO_4_, and concentrated in vacuo. The residue was further purified by silica gel column, and elution with 1%–5% MeOH in CH_2_Cl_2_ afforded the desired product **11** (30 mg, 36%, Rf = 0.4 in 5% MeOH/CH_2_Cl_2_) and **12** (25 mg, 28%, Rf = 0.2 in 5% MeOH/CH_2_Cl_2_) as red solid. Compound **12**: m.p. 213.4-216.5 °C. ^1^H-NMR (400 MHz, CDCl_3_) δ 8.99 (d, *J* = 10.5 Hz, 1H), 7.83 (d, *J* = 8.1 Hz, 1H), 7.75 (d, *J* = 8.3 Hz, 1H), 7.48 (s, 1H), 6.44 (d, *J* = 10.5 Hz, 1H), 4.71 (s, 2H), 1.52 (s, 6H). ^13^C-NMR (126 MHz, CDCl_3_) δ 201.6, 183.3, 175.5, 161.9, 151.6, 141.4, 138.8, 132.8, 132.5, 129.0, 128.0, 126.1, 125.2, 123.8, 120.1, 55.2, 48.1, 27.6. MS (ESI, [M + Na]^+^) *m*/*z* 345.2. HRMS (ESI, [M + H]^+^) calcd for C_19_H_15_O_5_, 323.0914; found, 323.0915.

##### Synthesis of 7-hydroxy-1,6,6-trimethyl-6,7-dihydrophenanthro[1,2-b]furan-10,11-dione (**10**) [33]

To a solution of **11** (30 mg, 0.098 mmol) in MeOH (2 mL) was added NaBH_4_ (11 mg, 0.300 mmol). The reaction mixture was stirred at rt for 1 h and then evaporated the solvent, diluted with H_2_O, and extracted with EtOAc (50 mL × 3). The combined organic layer was washed with brine, dried over anhydrous Na_2_SO_4_, and concentrated in vacuo. The residue was further purified by silica gel column, and elution with 1%–2% MeOH in CH_2_Cl_2_ afforded the desired product **10** (27 mg, 90%) as a red solid. m.p. 185.2-188.5 °C. ^1^H-NMR (400 MHz, CDCl_3_) δ 7.87 (d, *J* = 10.2 Hz, 1H), 7.64–7.55 (m, 2H), 7.24 (s, 1H), 6.39 (dd, *J* = 10.2, 4.4 Hz, 1H), 4.08 (d, *J* = 4.5 Hz, 1H), 2.26 (s, 3H), 1.39 (s, 3H), 1.28 (s, 3H). ^13^C-NMR (126 MHz, CDCl_3_) δ 184.2, 175.6, 161.2, 147.1, 141.5, 135.8, 135.5, 131.5, 127.7, 125.0, 123.6, 122.1, 121.2, 120.2, 72.5, 40.0, 26.3, 21.9, 8.8.

##### Synthesis of 1,6,6-trimethyl-7,8-dihydrophenanthro[1,2-b]furan-9,10,11(6*H*)-trione (**13**) and 1,6,6-trimethylphenanthro[1,2-b]furan-9,10,11(6*H*)-trione (**14**)

To a solution of **8** (200 mg, 0.644 mmol) in toluene (4 mL) and DMSO (2 mL) was added 2-Iodoxybenzoic acid (1.082 g, 3.864 mmol). The reaction mixture was stirred at 60 °C microwave for 3 h and then diluted with H_2_O and extracted with EtOAc (150 mL × 3). The combined organic layer was washed with brine, dried over anhydrous Na_2_SO_4_, and concentrated in vacuo. The residue was further purified by silica gel column, and elution with 30%–40% EtOAc in hexane afforded **13** (55 mg, 27%, Rf = 0.4 in 40% EtOAc/hexane) and **14** (38 mg, 19%, Rf = 0.2 in 40% EtOAc/hexane) as an orange solid. Compound **13**: ^1^H-NMR (300 MHz, CDCl_3_) δ 7.72 (d, *J* = 8.2 Hz, 1H), 7.57 (d, *J* = 8.2 Hz, 1H), 7.26 (d, *J* = 1.4 Hz, 1H), 2.92 (t, *J* = 7.2 Hz, 2H), 2.26 (d, *J* = 1.3 Hz, 3H), 2.07 (t, *J* = 7.2 Hz, 2H), 1.34 (s, 6H). Compound **14**: m.p. 158.3–160.8 °C.^1^H-NMR (400 MHz, CDCl_3_) δ 7.81 (d, *J* = 8.3 Hz, 1H), 7.72 (d, *J* = 8.3 Hz, 1H), 7.27 (s, 1H), 6.80 (d, *J* = 10.2 Hz, 1H), 6.48 (d, *J* = 10.2 Hz, 1H), 2.26 (s, 3H), 1.50 (s, 6H). ^13^C-NMR (126 MHz, CDCl_3_) δ 185.8, 183.4, 179.3, 159.6, 154.6, 151.8, 141.9, 135.3, 132.8, 131.6, 128.9, 127.4, 124.5, 121.3, 120.9, 38.3, 29.4, 8.7. MS (ESI, [M + H]^+^) *m*/*z* 307.4. HRMS (ESI) calcd for C_19_H_15_O_4_, 307.0965; found, 307.0967.

##### Synthesis of 1,6,6-trimethylphenanthro[1,2-b]furan-9,10,11(6*H*)-trione (**14**)

To a solution of **13** (44 mg, 0.14 mmol) in toluene (3 mL) was added 2,3-Dichloro-5,6-dicyano-1,4-benzoquinone (DDQ, 49 mg, 0.21 mmol). The reaction mixture was stirred at 80 °C for 36 h and then diluted with H_2_O and extracted with EtOAc (100 mL × 3). The combined organic layer was washed with brine, dried over anhydrous Na_2_SO_4_, and concentrated in vacuo. The residue was further purified by silica gel column, and elution with 40% EtOAc in hexane afforded **14** (31 mg, 70%).

##### Synthesis of 1-(hydroxymethyl)-6,6-dimethylphenanthro[1,2-b]furan-9,10,11(6*H*)-trione (**15**)

To a solution of **14** (30 mg, 0.098 mmol) in 1,4-dioxane (2 mL) was added selenium dioxide (44 mg, 0.392 mmol). The reaction mixture was stirred at 100 °C for 36 h and then diluted with H_2_O and extracted with EtOAc (50 mL × 3). The combined organic layer was washed with brine, dried over anhydrous Na_2_SO_4_, and concentrated in vacuo. The residue was further purified by silica gel column, and elution with 1%–2% MeOH in CH_2_Cl_2_ afforded the desired product **15** (5 mg, 16%) as an orange solid. m.p. 195.5–198.4 °C. ^1^H-NMR (400 MHz, CDCl_3_) δ 7.85 (d, *J* = 8.3 Hz, 1H), 7.75 (d, *J* = 8.3 Hz, 1H), 7.45 (s, 1H), 6.82 (d, *J* = 10.3 Hz, 1H), 6.47 (d, *J* = 10.2 Hz, 1H), 4.69 (s, 2H), 1.51 (s, 6H). ^13^C-NMR (126 MHz, CDCl_3_) δ 184.7, 183.3, 179.4, 161.1, 154.6, 152.5, 141.2, 135.7, 132.8, 131.8, 128.5, 127.4, 126.1, 124.7, 120.5, 55.2, 38.4, 29.5. HRMS (ESI, [M + H]^+^) calcd for C_19_H_15_O_5_, 323.0914; found, 323.0901.

### 3.2. Biology

hIDO-1 enzymatic assay. The hIDO-1 enzymatic assay was performed as described previously [36]. Briefly, a standard reaction mixture (30 μL) containing 100 mM potassium phosphate buffer (pH 6.5), 40 mmol/L ascorbic acid and 0.01% Triton X-100, 200 µg/mL catalase, 20 µmol/L methylene blue, and 0.05 µM rhIDO-1 was added to the solution (60 μL) containing the substrate l-tryptophan (250 µmol/L) and the test sample at a determined concentration. The reaction was carried out at 37 °C for 30 min and stopped by adding 45 µL of 30% (*w*/*v*) trichloroacetic acid. After being heated at 65 °C for 15 min, the reaction mixture was centrifuged at 12,000 rpm for 10 min. The supernatant (100 µL) was transferred into a well of a 96-well microplate and mixed with 100 µL of 2% (*w*/*v*) p-dimethylaminobenzaldehyde in acetic acid. The yellow pigment derived from kynurenine was measured at 492 nm using a Spectra Max Plus 384 microplate reader (Molecular Devices, Sunnyvale, CA, USA). IC_50_ values were calculated by using Graph Pad Prism 6 software (version 6.00, Graph Pad Software, San Diego, CA, USA).

Cell-based assay of IDO-1 activity. The cellular activity of IDO-1 was detected as described previously [36]. HEK 293 cells were seeded in a 6-well culture plate at a density of 5 × 10^5^ cells/well and cultured overnight. After 24 h, HEK 293 cells were transfected with pcDNA3.1-hIDO-1 using Lipofectamine 2000 according to the manufacturer’s instructions. Cells were seeded in a 96-well culture plate at a density of 2.5 × 10^4^ cells/well 24 h after transfection. A serial dilution of the tested compounds in 10 µL PBS was added to the cells. After an additional 12-h incubation, 200 µL of the supernatant per well was transferred to a new 96-well plate and mixed with 100 µL of 30% trichloroacetic acid in each well, and the plate was incubated at 65 °C for 15 min to hydrolyze *N*-formylkynurenine produced by the catalytic reaction of hIDO-1. The reaction mixture was then centrifuged for 10 min at 12,000 rpm to remove the sediments. Then, 100 µL of the supernatant per well were transferred to another 96-well plate and mixed with 100 µL of 2% (*w*/*v*) *p*-dimethylaminobenzaldehyde in acetic acid. The yellow color derived from kynurenine was measured at 492 nm using a Spectra Max Plus 384 microplate reader (Molecular Devices, Sunnyvale, CA, USA). IC_50_ values were calculated by using Graph Pad Prism 6 software (version 6.00, Graph Pad Software, San Diego, CA, USA).

### 3.3. Docking Study

Molecular modeling study of our compounds against IDO-1 was performed using the Schrödinger Suite 2015-4 (Schrödinger, LLC, New York, NY, USA). The X-ray crystal structure of IDO-1 was obtained from the Protein Data Bank. The IDO-1 protein preparation was revised using Protein Preparation Wizard. The receptor grid box was generated 25 × 25 × 25 Å cubic size. The compound was minimized using an OPLS_2005 force field with a dielectric constant value 80.0 in Macro Model. 

## 4. Conclusions

In conclusion, we have designed and synthesized a series of furano-*o*-naphthaquinone derivatives as novel IDO-1 inhibitors. Among them, 1-ene-3-ketone-17-hydroxyl derivative **12** exhibited significantly improved enzymatic and cellular inhibitory activity against IDO-1 when compared to acyloxy derivatives. Besides, the molecular docking study also disclosed compounds **12** and **11** have more interactions with the binding pocket of IDO-1 via hydrogen-bond, which may account for their higher IDO-1 inhibitory activity. Although our synthesized derivatives displayed relative lower inhibitory activity than reported IDO-1 inhibitors, the *o*-naphthaquinone scaffold together with the novel binding model will open new avenues to develop better IDO-1 inhibitors for the immuno-oncology therapy.

## Figures and Tables

**Table 1:** Structures of acyloxy derivatives **7a**–**m**.

Compound Number	R^1^	R^2^	Compound Number	R^1^	R^2^
**7a**	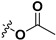	H	**7h**	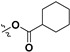	H
**7b**	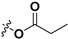	H	**7i**	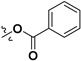	H
**7c**	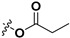	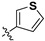	**7j**	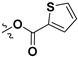	H
**7d**	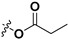	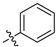	**7k**	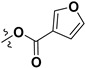	H
**7e**	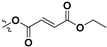	H	**7l**	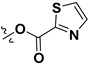	H
**7f**	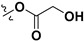	H	**7m**	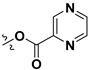	H
**7g**	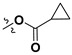	H			

**Table 2:** The IDO-1 inhibitory activity of *o*-naphthaquinone derivatives in enzyme- and cell-based assays.

Compound Number		R^1^	R^2^	Enzymatic IC_50_ (μM) or Inhibitory Rate at 20 µM ^a^	Cellular IC_50_ (μM) ^a^
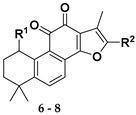	**6**	H	H	23.1 ± 1.6%	— ^b^
	**7a**	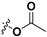	H	3.72 ± 0.45	—
	**7b**	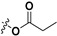	H	4.72 ± 0.30	—
	**7c**	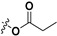	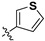	29.3 ± 2.2%	—
	**7d**	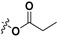	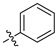	21.5 ± 1.1%	—
	**7e**	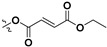	H	7.47 ± 3.58	—
	**7f**	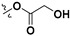	H	2.74 ± 0.10	—
	**7g**	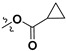	H	7.05 ± 2.46	—
	**7h**	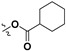	H	48.0 ± 3.2%	—
	**7i**	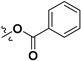	H	54.5 ± 2.3%	—
	**7j**	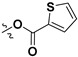	H	60.4 ± 3.9%	—
	**7k**	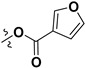	H	4.60 ± 2.81	—
	**7l**	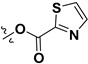	H	5.71 ± 0.47	—
	**7m**	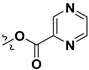	H	4.96 ± 2.02	—
	**8**	OH	H	2.83 ± 0.12	—
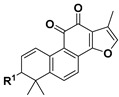	**9**	H		61.2 ± 2.1%	>10
	**10**	OH		3.10 ± 1.02	>10
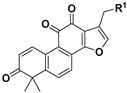	**11**	H		0.84 ± 0.26	>10
	**12**	OH		0.37 ± 0.02	3.85 ± 0.86
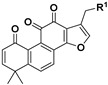	**14**	H		52.7 ± 2.5%	>10
	**15**	OH		68.0 ± 8.6%	—
Epacadostat				0.086 ± 0.009	0.023 ± 0.003

^a^ The values are the mean ± SE of two independent experiments; ^b^ Not determined.

## Supplementary Materials

The following are available online.
