# Supplementary material for: Discovery of Indoleamine 2,3-Dioxygenase 1 (IDO-1) Inhibitors Based on Ortho-Naphthaquinone-Containing Natural Product
Source: Molecules. 2019 Mar 18;24(6):1059. doi: 10.3390/molecules24061059 (PMC6471201; doi:10.3390/molecules24061059)
Supplement: Supplementary file 1 [file molecules-24-01059-s001.pdf]

Supplementary Material

Discovery of Indoleamine 2,3-Dioxygenase Inhibitors Based on Natural Product Tanshinone

Hongchuan Zhao [1,#], Pu Sun [2,#] Wei Guo [2] Yi Wang [2] Ao Zhang [1,3,4] Linghua Meng [*,2], and Chunyong Ding [*,1,4]

[1] CAS Key Laboratory of Receptor Research, State Key Laboratory of Drug Research, Synthetic Organic & Medicinal Chemistry Laboratory, Shanghai Institute of *Materia Medica*, Chinese Academy of Sciences, Shanghai 201203, China

[2] Division of Anti-tumor Pharmacology, Shanghai Institute of *Materia Medica*, Chinese Academy of Sciences, Shanghai 201203, China

[3] ShanghaiTech University, Shanghai 20120, China

[4] University of Chinese Academy of Sciences, Beijing 100049, China.

[#] These authors contributed equally to this work

* Correspondence and requests for materials should be addressed to C. D. (email: chding@simm.ac.cn), or L. M. (lhmeng@simm.ac.cn)

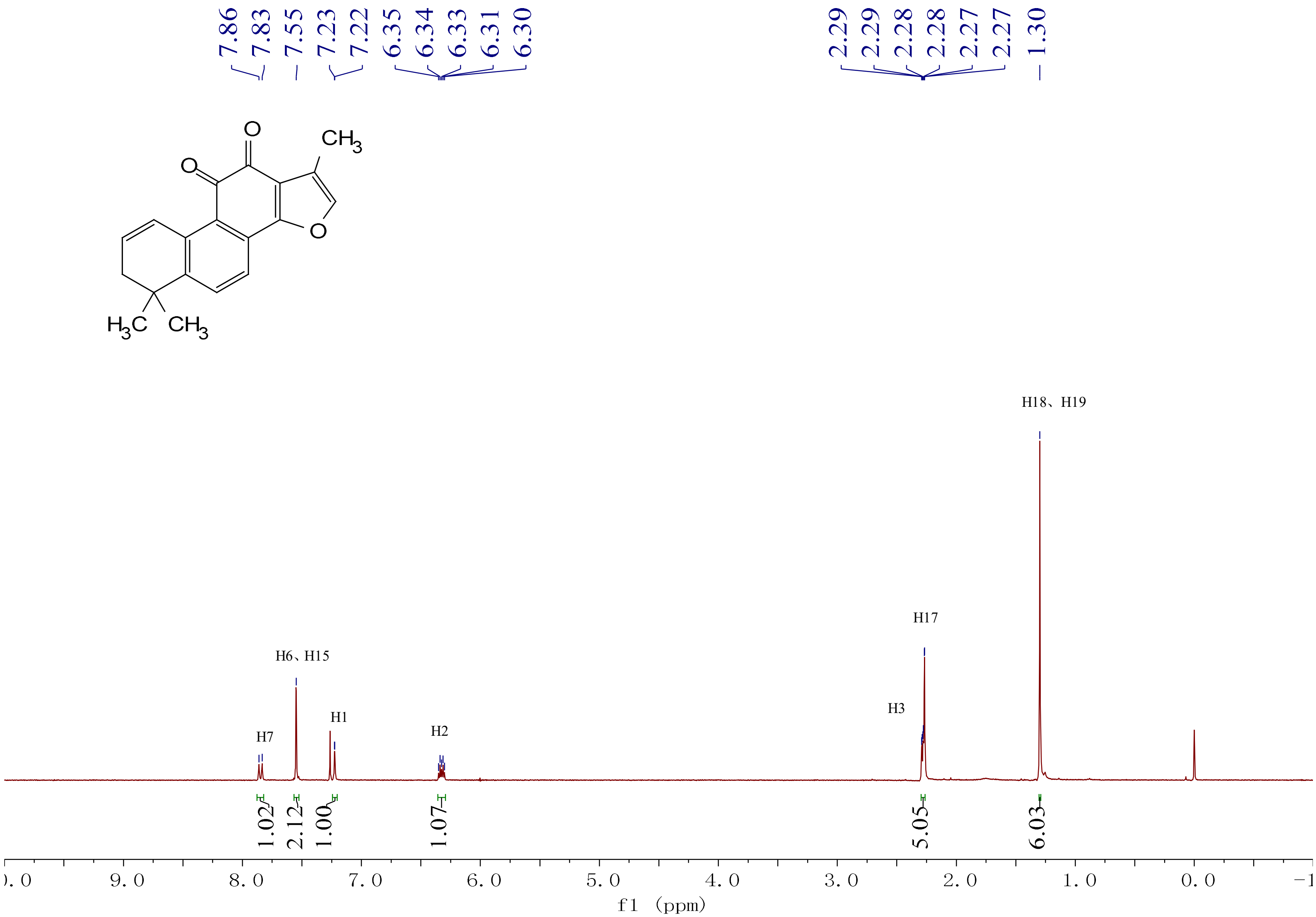

Figure S1. ^{1}H-NMR spectrum of compound **9**

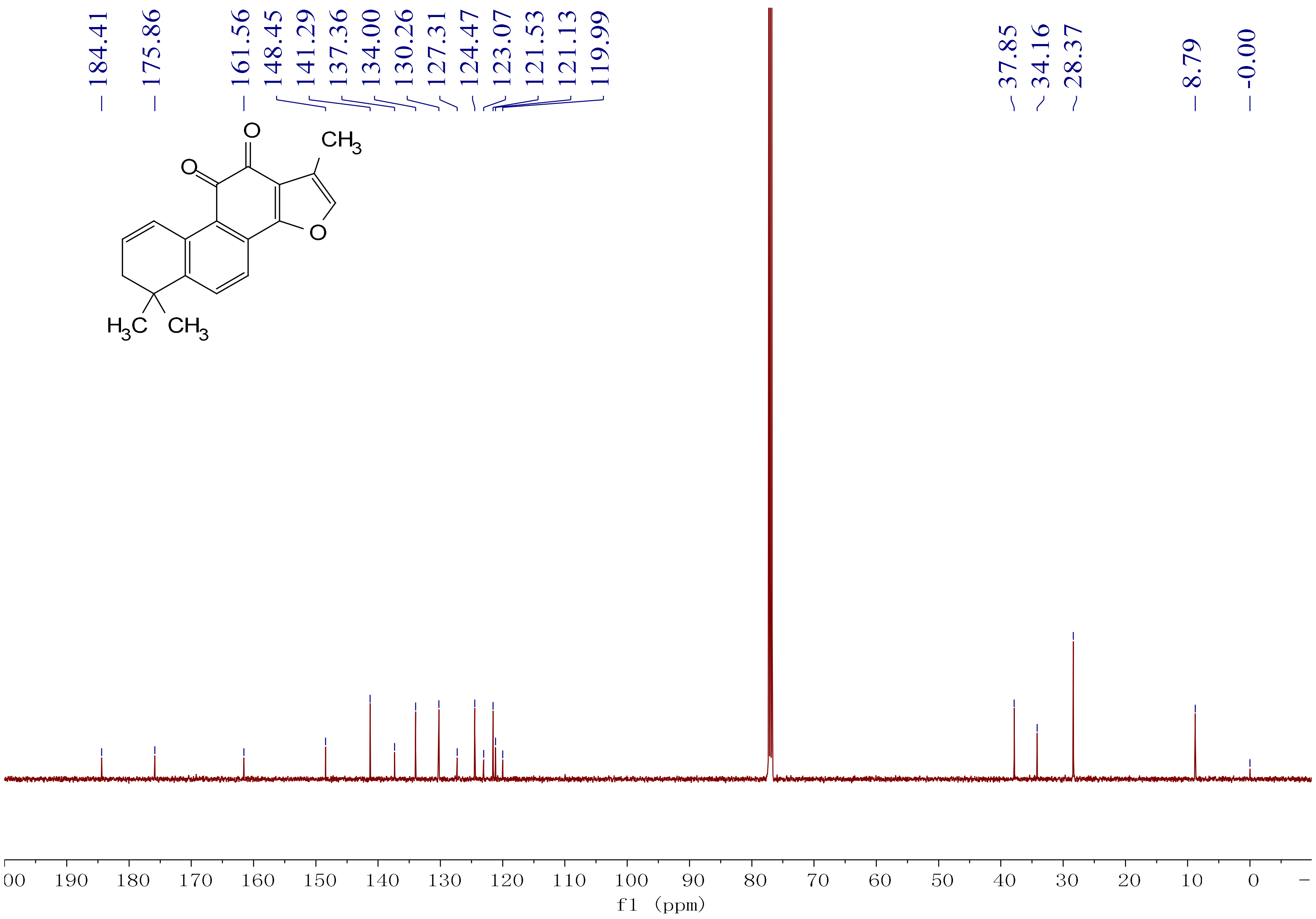

Figure S2. ^{13}C-NMR spectrum of compound **9**

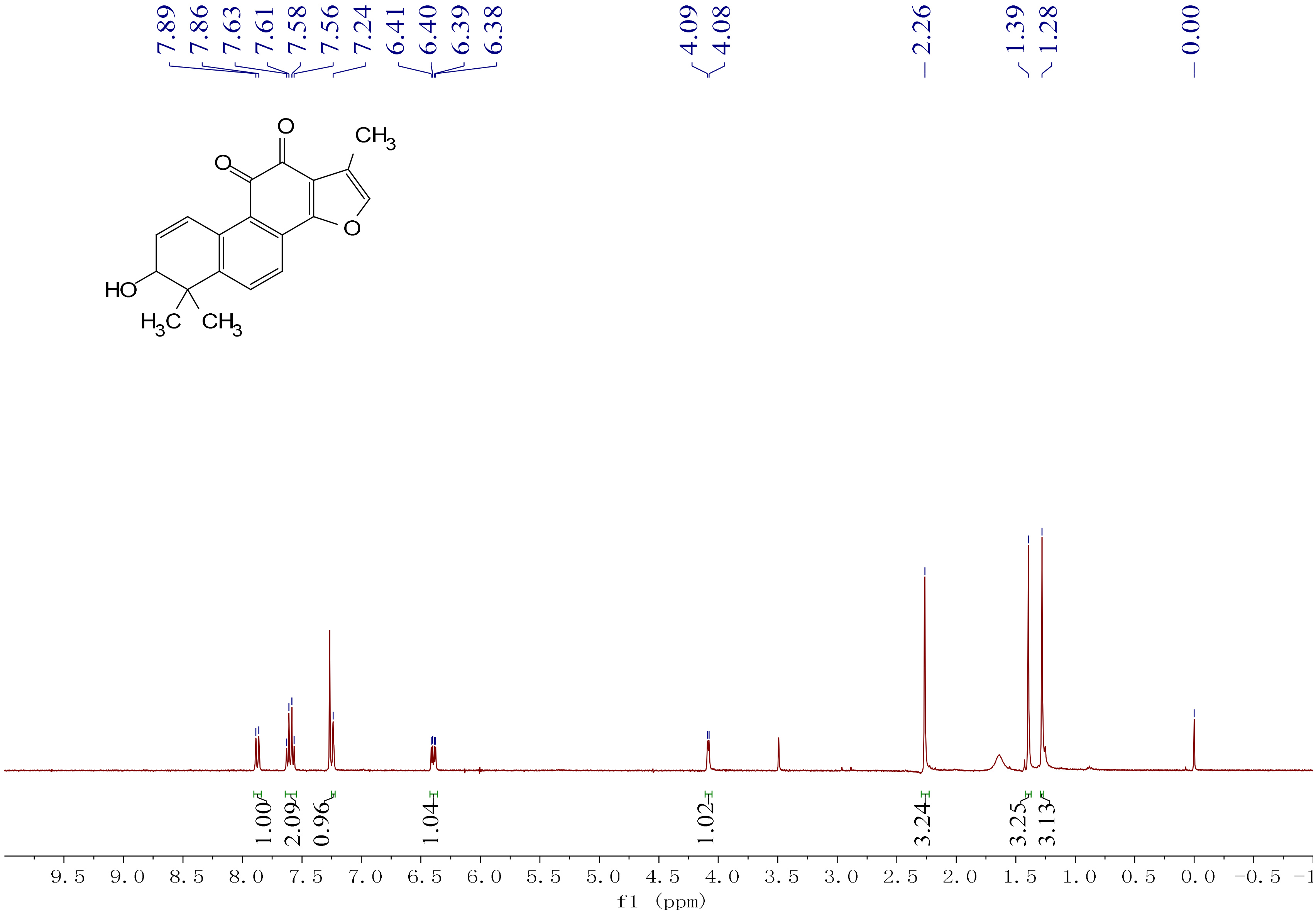

Figure S3. ^{1}H-NMR spectrum of compound **10**

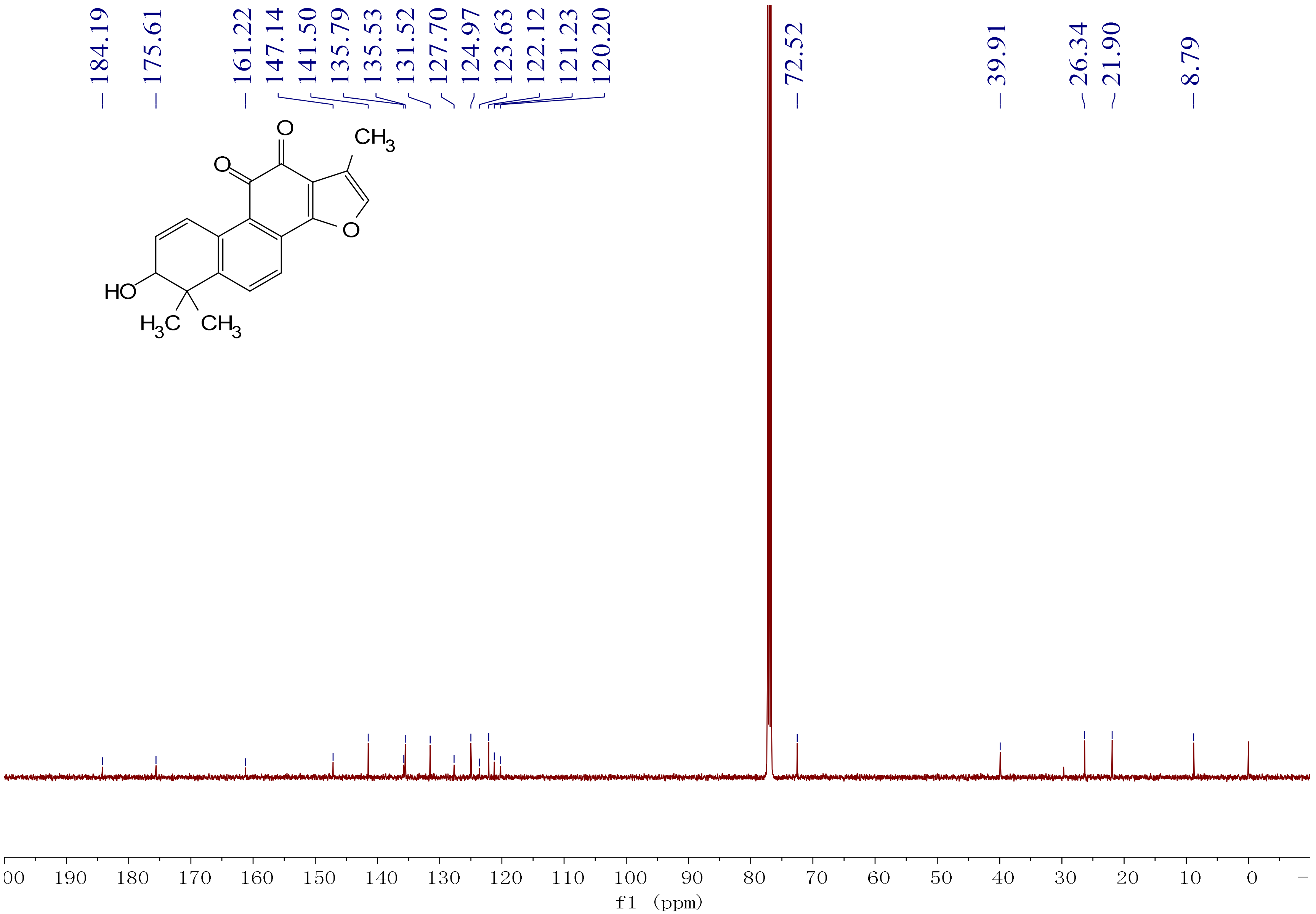

Figure S4. ^{13}C-NMR spectrum of compound **10**

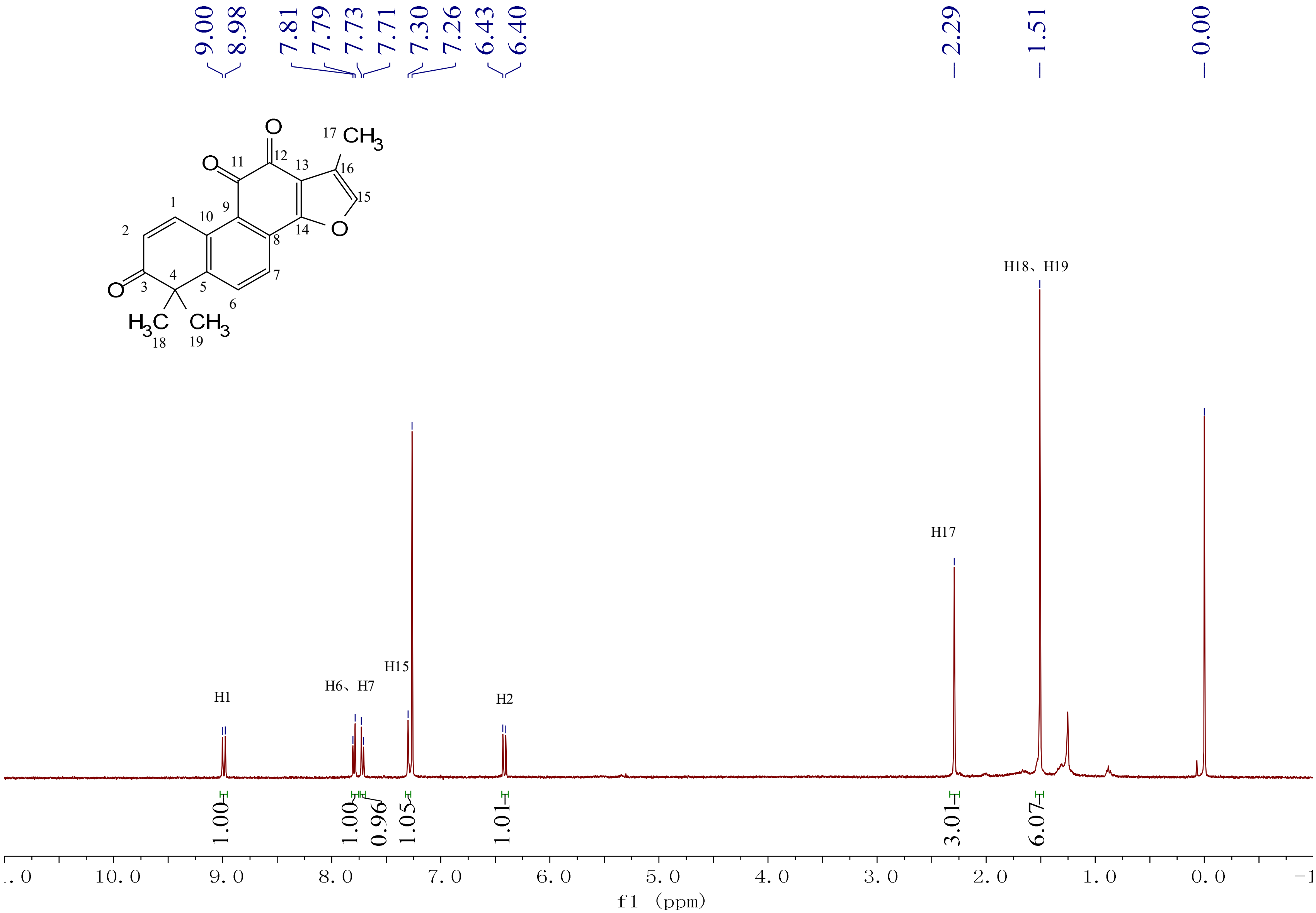

Figure S5. ^{1}H-NMR spectrum of compound **11**

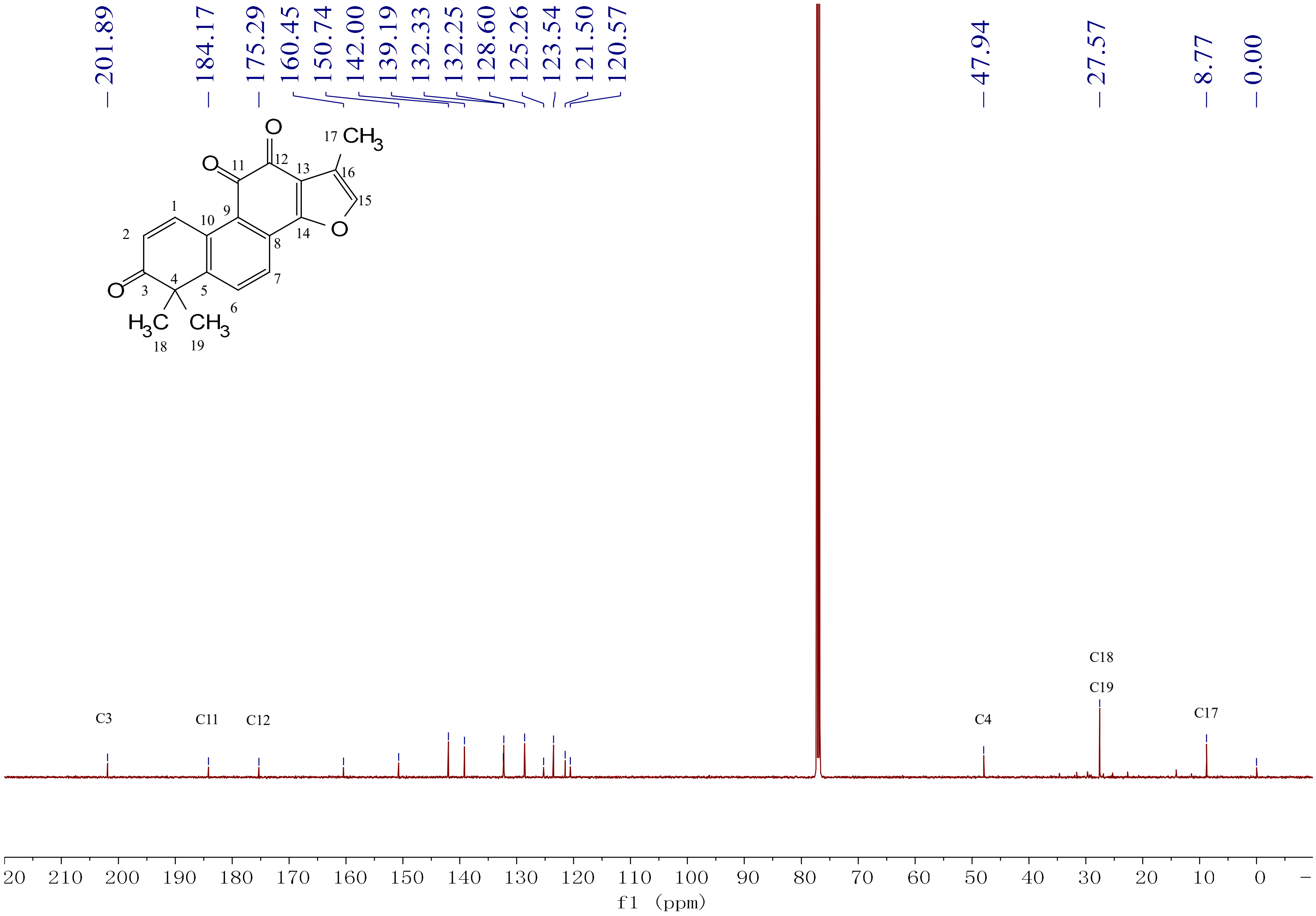

Figure S6. ^{13}C-NMR spectrum of compound **11**

Qualitative Analysis Report

**Data Filename**	ESIH_20181226_ZA_ZHC_04.d	**Sample Name**	D5-12598-024
**Sample Type**	Sample	**Position**	P1-A1
**Instrument Name**	Agilent G6520 Q-TOF	**Acq Method**	20160322_MS_ESIH_POS_1min.m
**Acquired Time**	12/26/2018 13:25:43	**IRM Calibration Status**	Success
**DA Method**	small molecular data analysis method.m	**Comment**	ESIH by ZZY

User Spectra

Fragmentor Voltage	Collision Energy	Ionization Mode
125	0	ESI

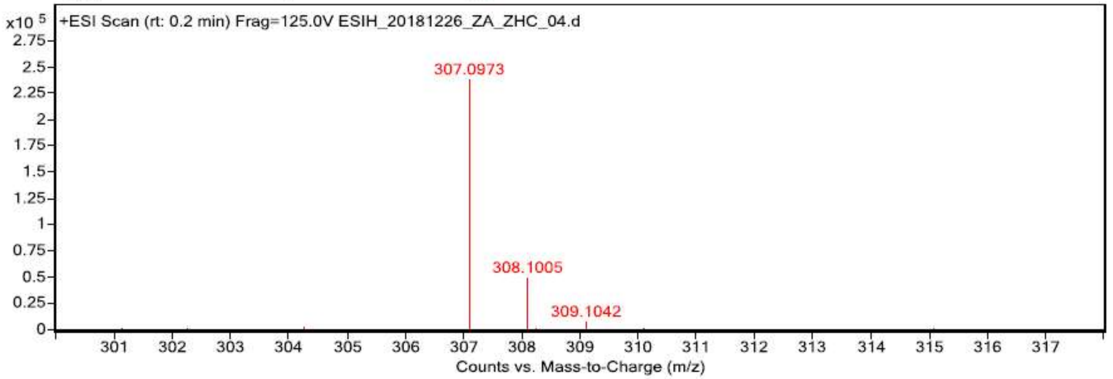

Formula Calculator Results

m/z	Calc m/z	Diff (mDa)	Diff (ppm)	Ion Formula	Ion
307.0973	307.0965	-0.77	-2.51	C19 H15 O4	(M+H)+

--- End Of Report ---

Figure S7. HRMS of compound **11**

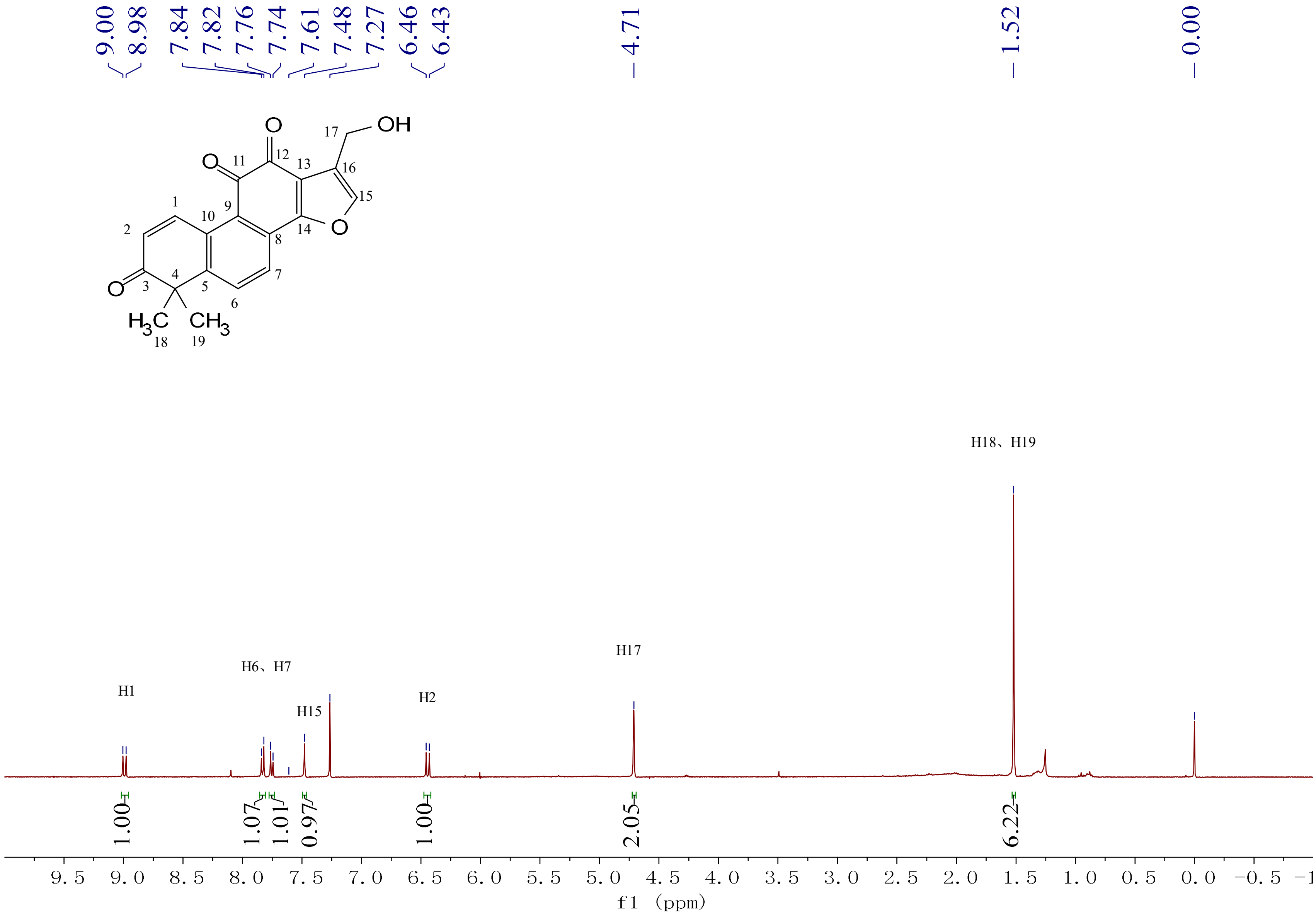

Figure S8. ^{1}H-NMR spectrum of compound **12**

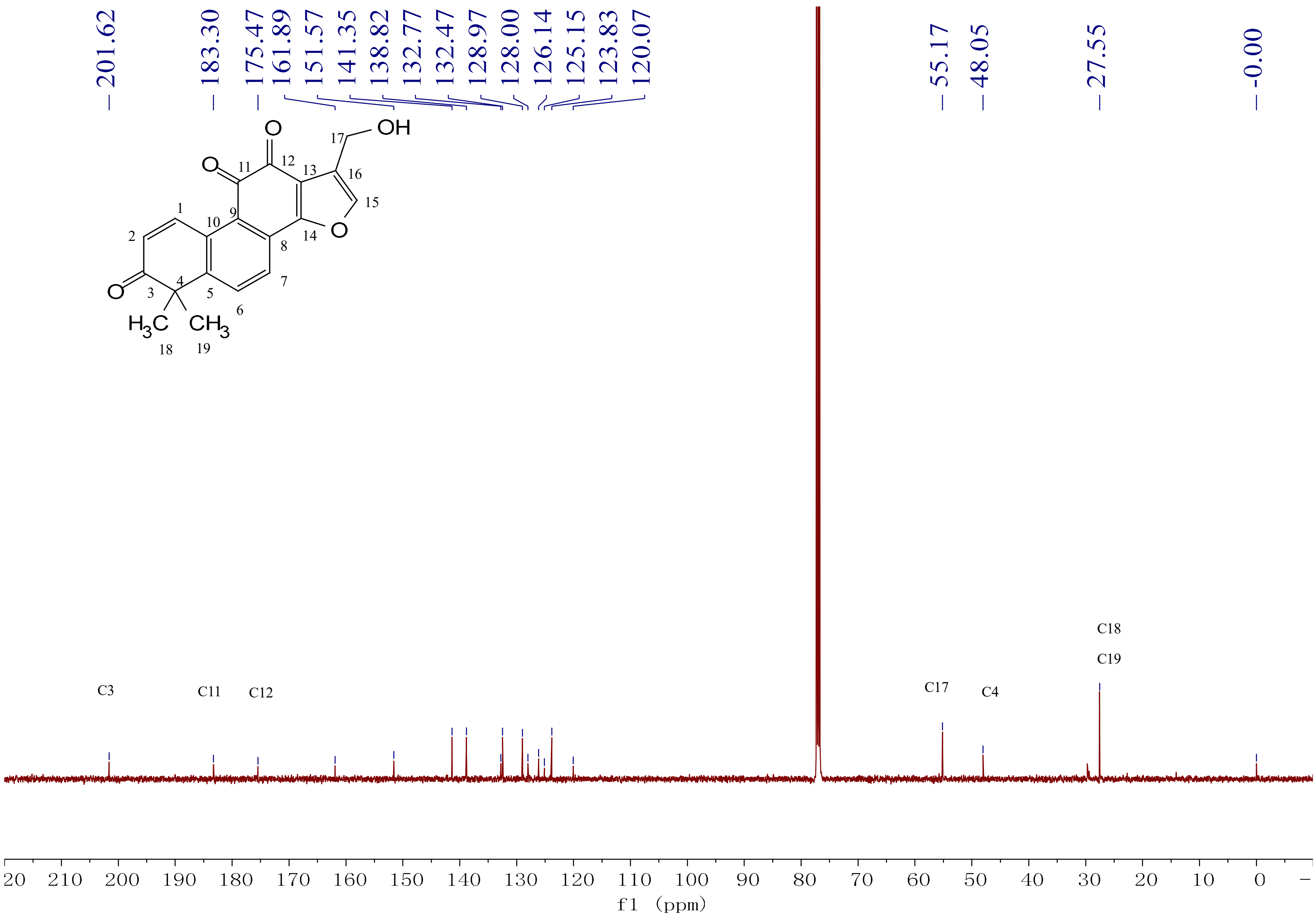

Figure S9. ^{13}C-NMR spectrum of compound **12**

Qualitative Analysis Report

**Data Filename**	ESIH_20181226_ZA_ZHC_05.d	**Sample Name**	D5-12598-044
**Sample Type**	Sample	**Position**	P1-A2
**Instrument Name**	Agilent G6520 Q-TOF	**Acq Method**	20160322_MS_ESIH_POS_1min.m
**Acquired Time**	12/26/2018 13:27:43	**IRM Calibration Status**	Success
**DA Method**	small molecular data analysis method.m	**Comment**	ESIH by ZZY

User Spectra

Fragmentor Voltage	Collision Energy	Ionization Mode
125	0	ESI

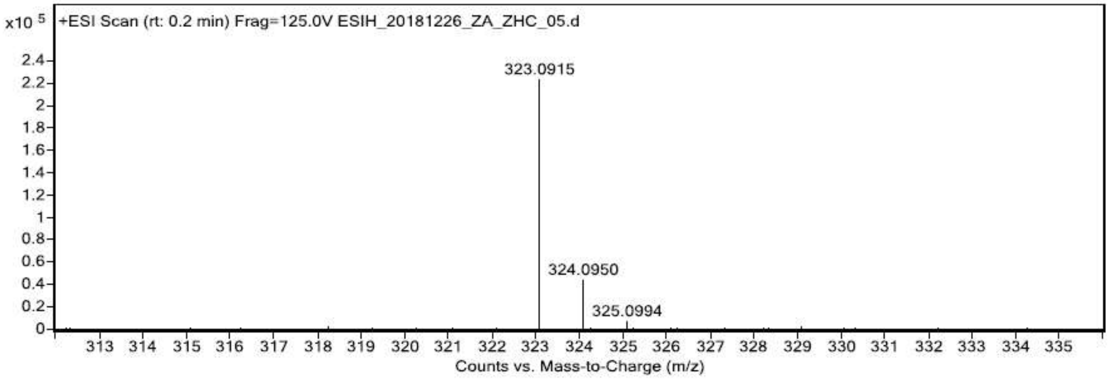

Formula Calculator Results

m/z	Calc m/z	Diff (mDa)	Diff (ppm)	Ion Formula	Ion
323.0915	323.0914	-0.07	-0.21	C19 H15 O5	(M+H)+

--- End Of Report ---

Figure S10. HRMS of compound **12**

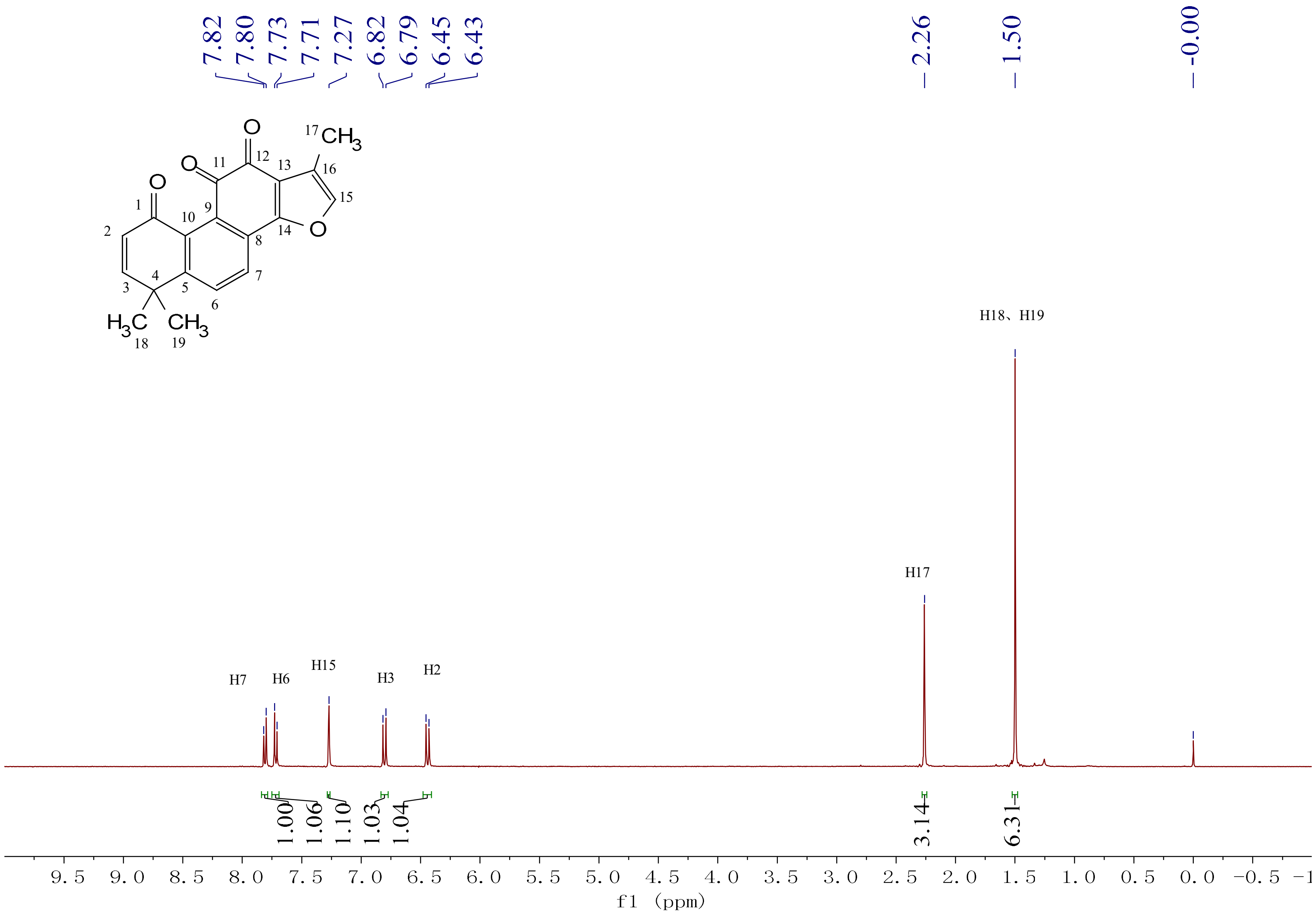

Figure S11. ^{1}H-NMR spectrum of compound **14**

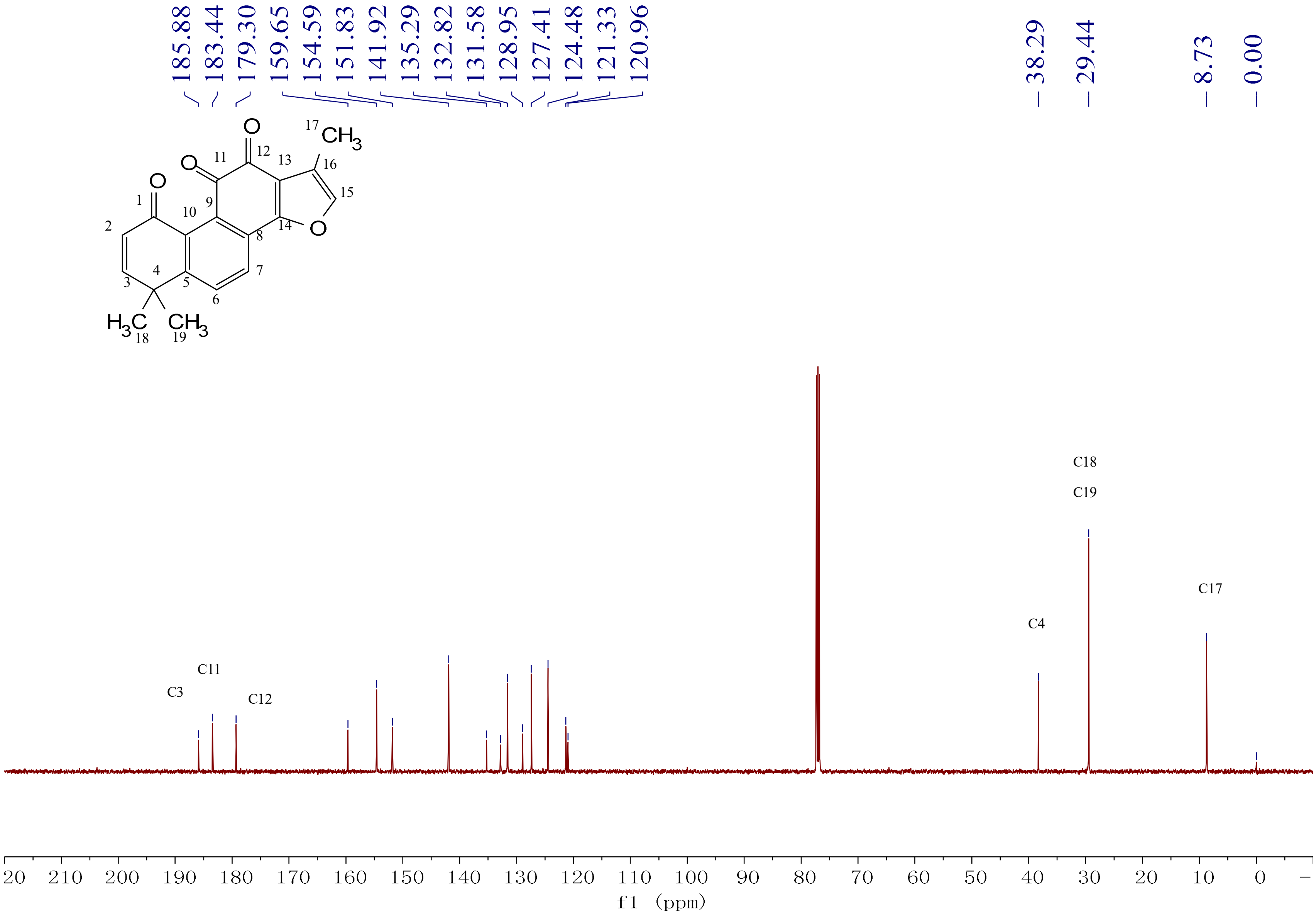

Figure S12. ^{13}C-NMR spectrum of compound **14**

Qualitative Analysis Report

**Data Filename**	ESIH_20181226_ZA_ZHC_02.d	**Sample Name**	D5-12598-501
**Sample Type**	Sample	**Position**	P1-A1
**Instrument Name**	Agilent G6520 Q-TOF	**Acq Method**	20160322_MS_ESIH_POS_1min.m
**Acquired Time**	12/26/2018 13:20:22	**IRM Calibration Status**	Success
**DA Method**	small molecular data analysis method.m	**Comment**	ESIH by ZZY

User Spectra

Fragmentor Voltage	Collision Energy	Ionization Mode
125	0	ESI

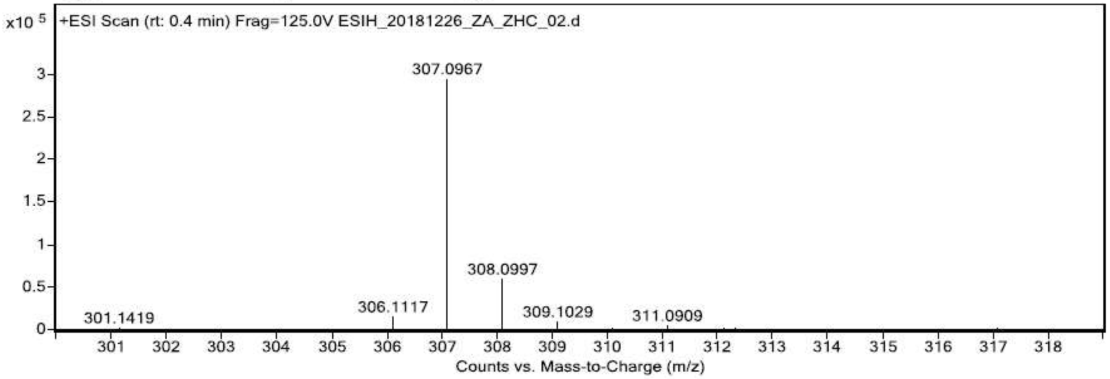

Formula Calculator Results

m/z	Calc m/z	Diff (mDa)	Diff (ppm)	Ion Formula	Ion
307.0967	307.0965	-0.24	-0.77	C19 H15 O4	(M+H)+

--- End Of Report ---

Figure S13. HRMS of compound **14**

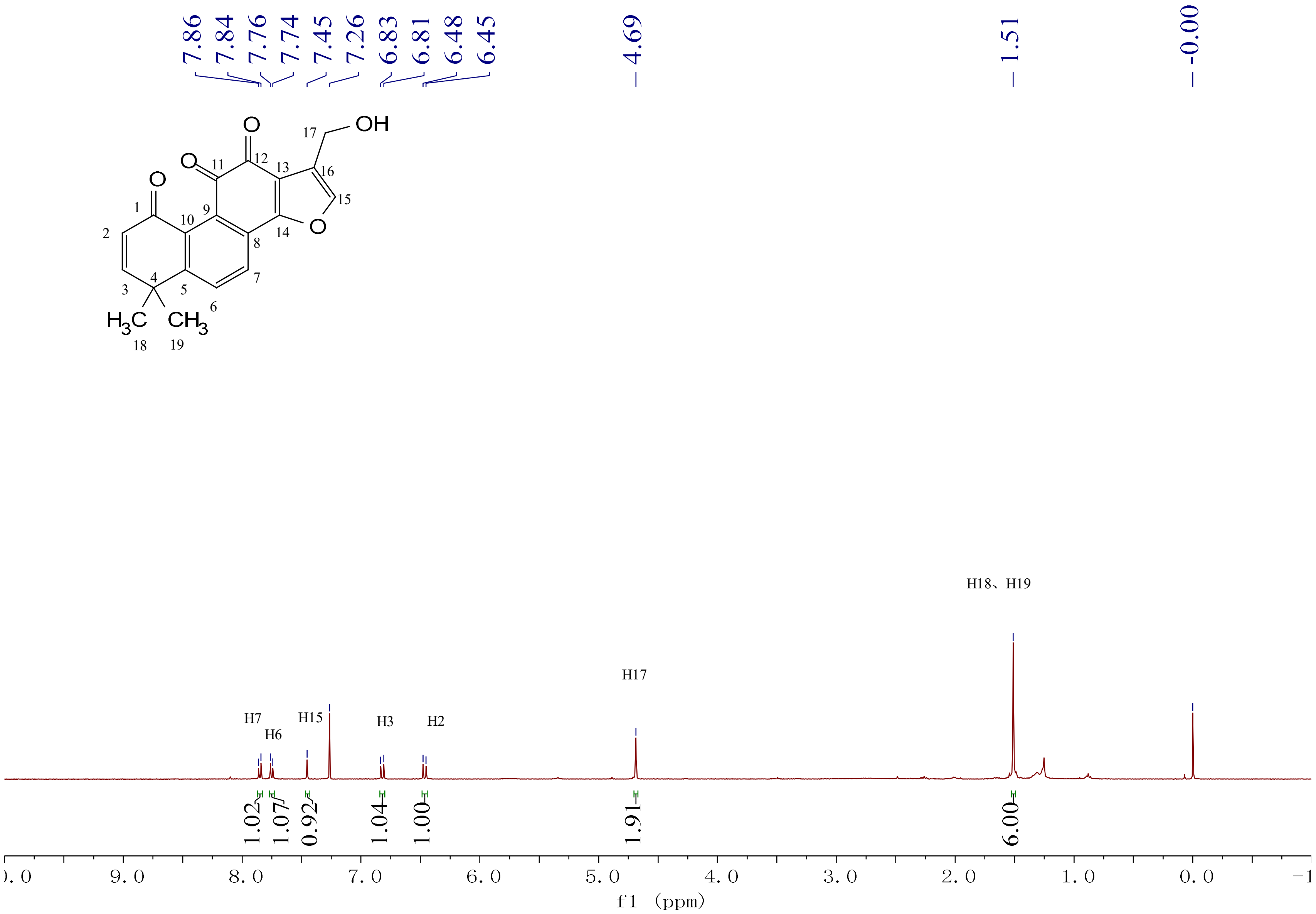

Figure S14. ^{1}H-NMR spectrum of compound **15**

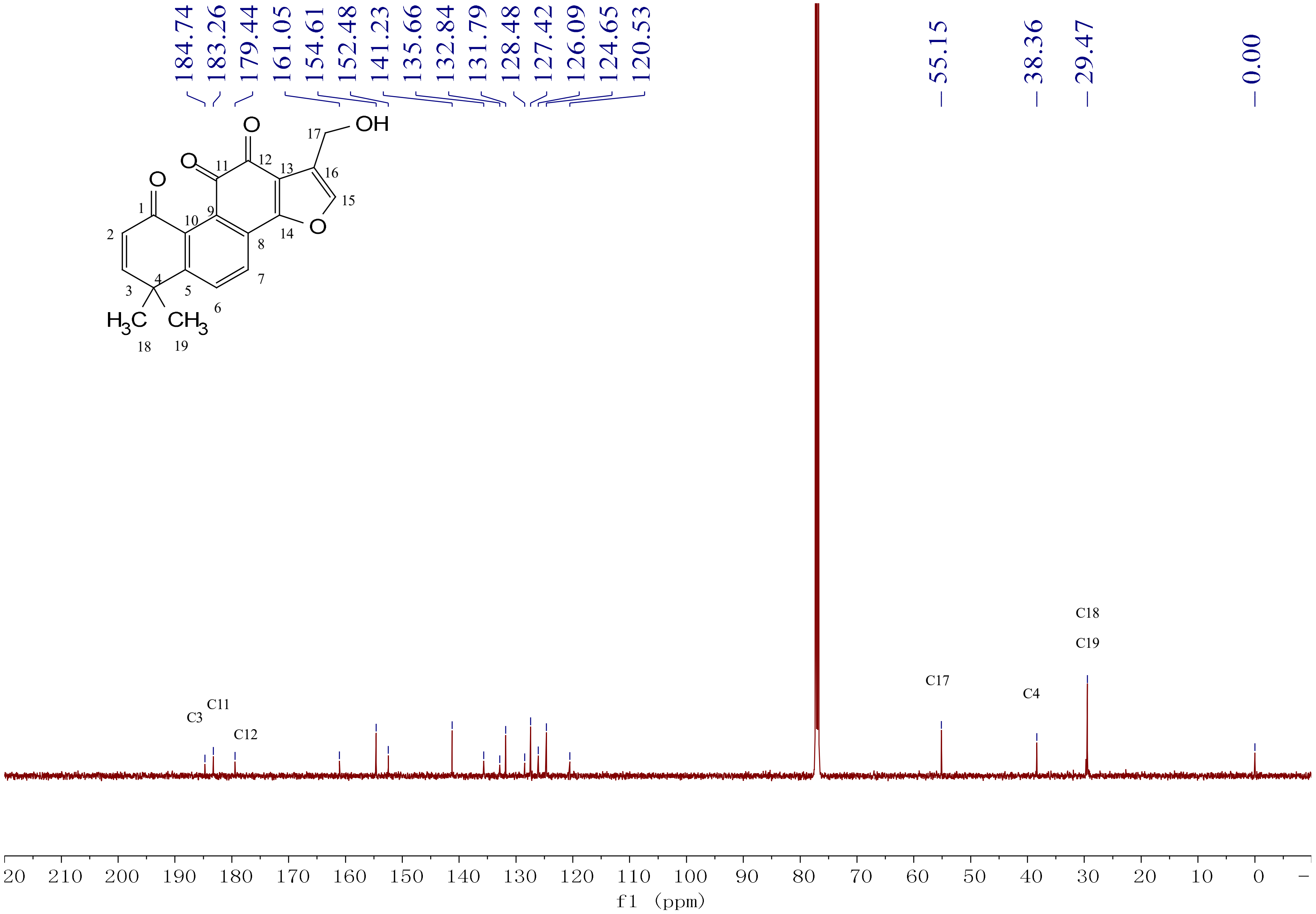

Figure S15. ^{13}C-NMR spectrum of compound **15**

Qualitative Analysis Report

**Data Filename**	ESIH_20181226_ZA_ZHC_03.d	**Sample Name**	D5-12598-502
**Sample Type**	Sample	**Position**	P1-A2
**Instrument Name**	Agilent G6520 Q-TOF	**Acq Method**	20160322_MS_ESIH_POS_1min.m
**Acquired Time**	12/26/2018 13:22:25	**IRM Calibration Status**	Success
**DA Method**	small molecular data analysis method.m	**Comment**	ESIH by ZZY

User Spectra

Fragmentor Voltage	Collision Energy	Ionization Mode
125	0	ESI

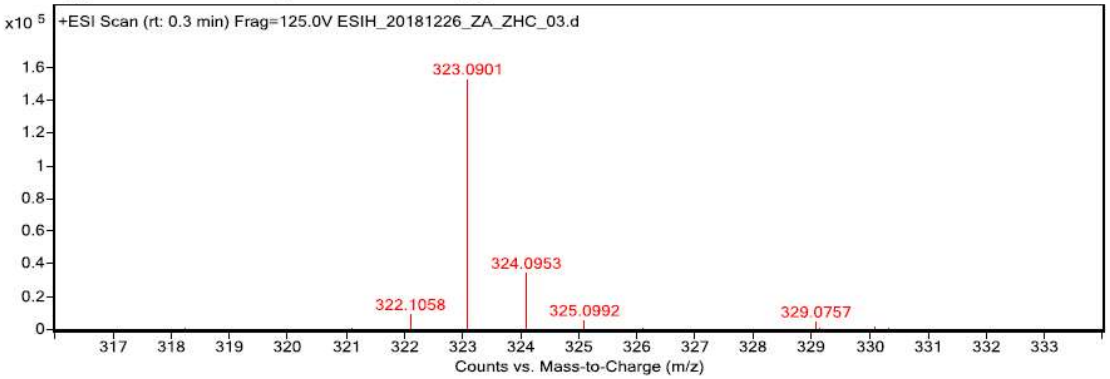

Formula Calculator Results

m/z	Calc m/z	Diff (mDa)	Diff (ppm)	Ion Formula	Ion
323.0901	323.0914	1.29	3.98	C19 H15 O5	(M+H)+

--- End Of Report ---

Figure S16. HRMS of compound **15**

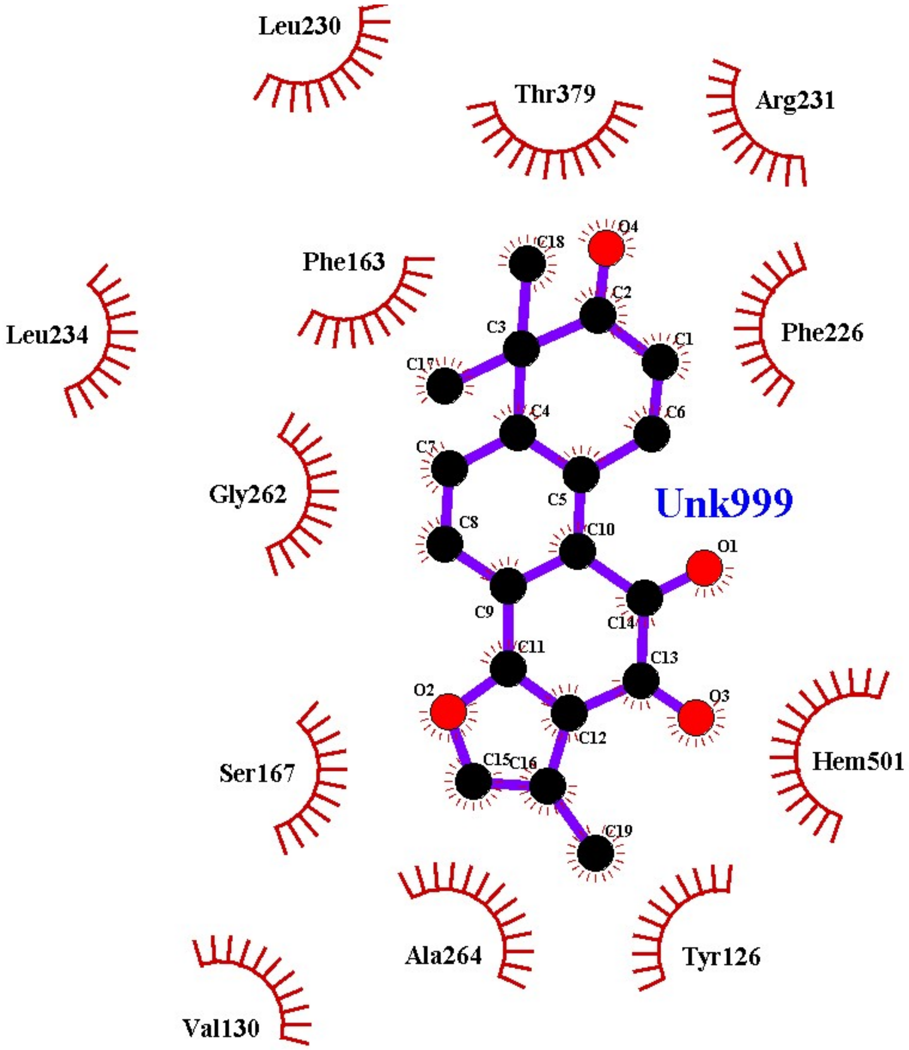
Leu230
Thr379
Arg231
Phe163
Leu234
Phe226
Gly262
Unk999
Ser167
Hem501
Ala264
Tyr126
Val130
C18
O4
C2
C3
C1
C17
C4
C6
C7
C5
C10
C8
O1
C9
C14
C11
C13
O2
O3
C12
C15
C16
C19

（a）

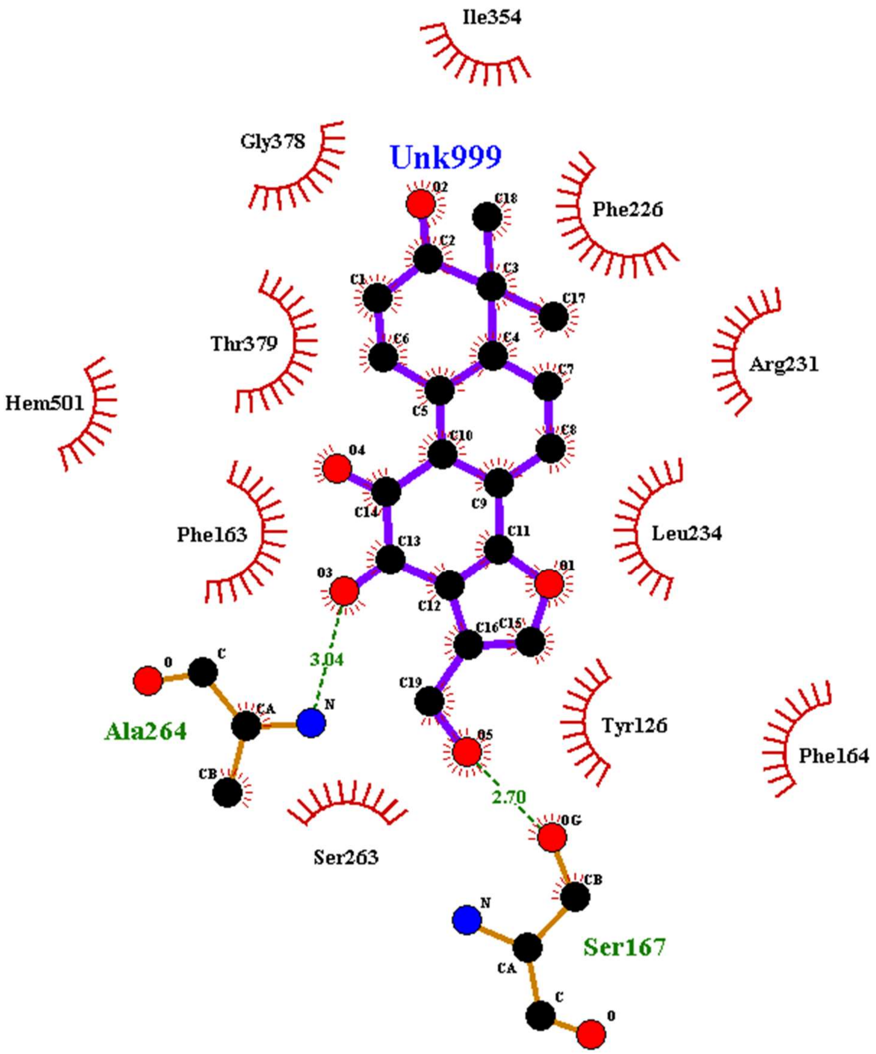
Ile354
Gly378
Unk999
Phe226
Thr379
Arg231
Hem501
Phe163
Leu234
3.04
Ala264
Tyr126
Phe164
2.70
Ser263
Ser167
O2
C18
C2
C3
C1
C17
C6
C4
C7
C5
C10
C8
O4
C14
C9
C13
C11
O3
O1
C12
C16
C15
C19
O5
OG
CB
N
CA
C
O

（b）

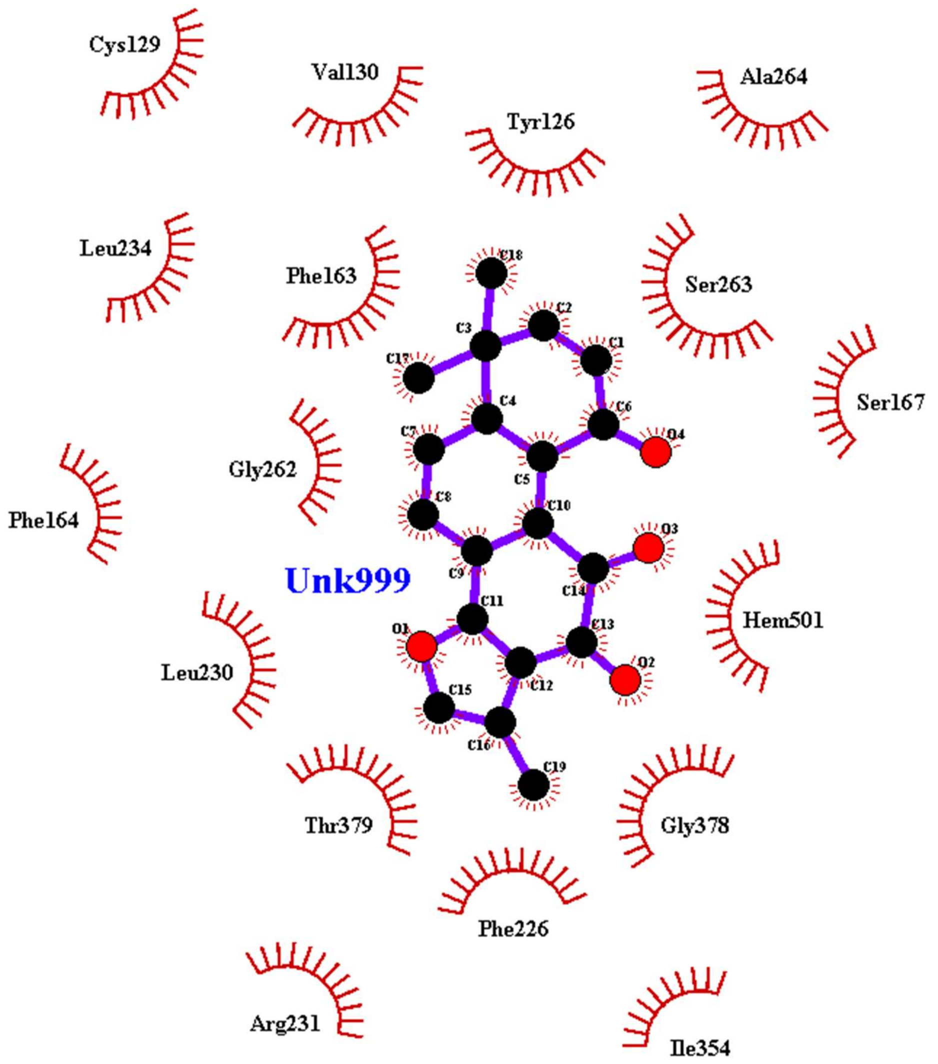

(c)

Figure S17. (**a**) 2D binding mode of **11** against IDO-1; (**b**) 2D binding mode of **12** against IDO-1; (**c**) 2D binding mode of **14** against IDO-1.
